# Differences in health-related quality of life between native and foreign-born gynaecological cancer patients in Sweden: a five-year cross-sectional study

**DOI:** 10.1007/s11136-023-03548-1

**Published:** 2023-11-06

**Authors:** Andreas Karlsson Rosenblad, Bodil Westman, Karin Bergkvist, Ralf Segersvärd, Nathalie Roos, Mia Bergenmar, Lena Sharp

**Affiliations:** 1 Regional Cancer Centre Stockholm-Gotland, Box 6909, SE-102 39 Stockholm, Sweden; 2 Division of Clinical Diabetology and Metabolism, Department of Medical Sciences, Uppsala University, Uppsala, Sweden; 3 Division of Family Medicine and Primary Care, Department of Neurobiology, Care Sciences and Society, Karolinska Institutet, Stockholm, Sweden; 4 Department of Statistics, Uppsala University, Uppsala, Sweden; 5 Department of Care Science, Sophiahemmet University, Stockholm, Sweden; 6 Division of Nursing, Department of Neurobiology, Care Sciences and Society, Karolinska Institutet, Stockholm, Sweden; 7 Department of Surgery, CLINTEC, Karolinska Institutet, Stockholm, Sweden; 8 Division of Clinical Epidemiology, Department of Medicine, Karolinska Institutet, Stockholm, Sweden; 9 Department of Oncology-Pathology, Karolinska Institutet, Stockholm, Sweden; 10 Department of Nursing, Umeå University, Umeå, Sweden

**Keywords:** Demography, Foreign-born, Gynaecological cancer, Health-related quality of life, Socioeconomic factors, Women

## Abstract

**Purpose:**

To examine differences in health-related quality of life (HRQoL) between native and foreign-born gynaecological cancer patients in Sweden, taking into account clinical, demographic, and socioeconomic factors.

**Methods:**

The 30-item European Organisation for Research and Treatment of Cancer quality of life questionnaire (QLQ-C30) and a study-specific questionnaire covering demographic and socioeconomic factors were answered by 684 women aged ≥ 18 years old, diagnosed in 2014, 2016, or 2018 with gynaecological cancer in the Stockholm-Gotland health care region, Sweden. Clinical data were obtained from the Swedish Cancer Register. Data were analysed using the Kruskal–Wallis test and linear regression.

**Results:**

The women had a mean age of 65.4 years, with 555 (81.1%) born in Sweden, 54 (7.9%) in other Nordic countries (ONC), 43 (6.3%) in other European countries (OEC), and 32 (4.7%) in non-European countries (NEC). HRQoL differed significantly between the four groups for 14 of the 15 QLQ-C30 scales/items. On average, Swedish-born women scored 2.0, 15.2, and 16.7 points higher for QoL/functioning scales/items and 2.2, 14.1, and 18.7 points lower for symptom scales/items, compared with ONC-, OEC-, and NEC-born women, respectively. In adjusted analyses, none of the differences between Swedish-born and ONC-born women were significant, while for OEC- and NEC-born women the differences were significant for most QLQ-C30 scales/items.

**Conclusion:**

HRQoL differs between native and foreign-born gynaecological cancer patients in Sweden, with lower HRQoL the further from Sweden the women are born. A more individualised cancer care, with tailored support to optimize HRQoL is needed for this vulnerable group of patients.

## Introduction

Cancer is a leading cause of death worldwide, accounting for almost 10 million deaths yearly. Among women, there are 4.4 million cancer-related deaths globally each year, with breast cancer being the most common cause of death, accounting for 15.5% of all deaths, or about 685,000 deaths each year. The five major gynaecological cancers (cervical, uterine, ovarian, vulvar, and vaginal cancer) are causing 670,000 deaths each year, or about 15.2% of all female cancer-related deaths. Taken together, these cancers constitute the second most common cause of cancer-related death among women. [1] In Sweden, cancer is the second most common cause of death among women, with a total of 11,205 deaths in 2021, corresponding to 24.6% of all deaths among women, of which 1198 (10.7%) were caused by gynaecological cancers [2]. With almost 3000 women being diagnosed with gynaecological cancers in Sweden yearly, the number of women living with gynaecological cancer in Sweden is increasing rapidly [3].

With an increasing number of persons living with cancer, the importance of cancer patients’ health-related quality of life (HRQoL), a multi-dimensional concept commonly used to examine the impact of health status on quality of life (QoL), is increasing. HRQoL is a patient-reported outcome measure (PROM) known to be affected by both the disease itself and the cancer treatment. Knowledge of patients’ HRQoL is thus important for meeting and understanding patients' needs in relation to the severity and progression of the disease as well as the treatment they are given [4, 5]. In addition to clinical factors, such as tumour size and disease stage, previous studies have indicated that socioeconomic and demographic factors such as age, employment status, education level, and country of birth may be related to cancer patients’ HRQoL [5–10]. However, research in this area is scarce, with small study populations and questionable generalizability of the results.

In Sweden, the percentage of foreign-born residents has almost doubled during the last couple of decades, from 11.3% in 2000 to 20.0% in 2021, equalling approximately 2.1 million people. The most populous Swedish region, the capital region of Stockholm with a population of 2.4 million people, has the largest proportion of foreign-born inhabitants, amounting to 26.5% of the inhabitants in 2021 [11]. The question of possible differences in HRQoL between native and foreign-born cancer patients in Sweden is thus of increasing importance. Moreover, it is known that foreign-born women are disadvantaged compared to foreign-born men, having a considerably lower employment rate, lower education level, and more often having health problems, [12] making this vulnerable group particularly important to study.

### Aim

The aim of the present study was to examine differences in HRQoL between native and foreign-born gynaecological cancer patients in Sweden, taking into account clinical, demographic, and socioeconomic factors.

## Materials and methods

The present study is a secondary analysis of data from a study intended to evaluate the newly introduced role of coordinating contact nurses, implemented in cancer care in the Stockholm-Gotland health care region (HCR) during the 2010s [13]. Stockholm-Gotland is one of six HCRs in Sweden, consisting of the two regional councils Region Stockholm and Region Gotland. Results from the latter study, which besides gynaecological cancer patients also included patients diagnosed with haematological, head and neck, or upper gastro-intestinal cancers, have been published elsewhere [13–17].

### Study design and data collection

Patients aged ≥ 18 years old diagnosed in the Stockholm-Gotland HCR with vulvar, vaginal, cervical, uterine, ovarian, or fallopian tube cancer (International Statistical Classification of Diseases and Related Health Problems, 10th revision [ICD-10] codes C51–C54, C559, C569, or C570) during 2014, 2016, or 2018 were identified using the Swedish Cancer Register (SCR). Reporting to the SCR, maintained by the Swedish National Board of Health and Welfare, is mandated by law, and the SCR thus includes approximately 99% of all cancer cases in Sweden [18]. Postal addresses to each patient, together with information on whether they were still alive, were obtained from the Swedish Population Register. Those with a valid address who were still alive were deemed eligible to participate in the study.

A letter containing information about the purpose of the study and an invitation to participate was sent by regular mail to patients deemed eligible to participate. The invitation included a study-specific questionnaire, the Swedish language version of the 30-item European Organisation for Research and Treatment of Cancer (EORTC) quality of life questionnaire (QLQ-C30) [19–21], and a pre-stamped envelope for returning the questionnaire. A secure internet link was also provided for those who preferred to complete the questionnaire online.

The patients were informed that participation was voluntary, and that non-participation would not affect their future care. Details on how to obtain additional information about the study from the research team were also provided. Responding to the questionnaire was considered as consenting to participate in the study. Patients who did not intend to participate were asked to return a blank copy of the questionnaire. One reminder was sent to non-responding patients about three weeks after the first invitation.

**Fig. 1:**
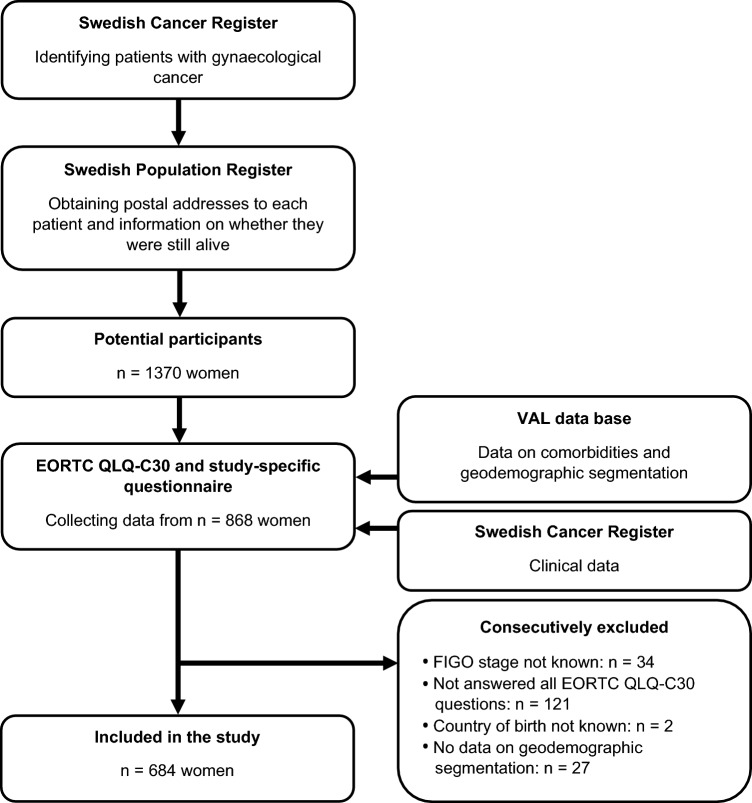
Flowchart of the data collection and inclusion process

After collecting the patient-reported questionnaire data, additional data on clinical characteristics such as FIGO stage, comorbidities, and geodemographic segmentation were obtained from the SCR and the VAL (*Vårdadministrativt datalager*, Healthcare administrative data warehouse) data base maintained by Region Stockholm through linkage using each patient’s unique Swedish Personal Identification Number (PIN). A flowchart describing the data collection and inclusion process is presented in Fig. 1.

### Study population

For the present secondary analysis study, we identified 1370 women who had been diagnosed with gynaecological cancer in the Stockholm-Gotland HCR during 2014, 2016, or 2018, of which 868 (63.4%) had responded to the study-specific questionnaire. Of these 868 potentially eligible participants, 34 women without known FIGO stage, 121 women who had not answered all EORTC QLQ-C30 questions, 2 women without known country of birth, and 27 women without data on geodemographic segmentation were consecutively excluded, resulting in a study population of 684 women (Fig. 1). Notably, since data on geodemographic segmentation were available only for those living in Region Stockholm, no women living in Region Gotland were included in the present study.

### The European organisation for research and treatment of cancer quality of life questionnaire (EORTC QLQ-C30)

EORTC QLQ-C30 is a well-established instrument designed for measuring HRQoL among cancer patients [16]. It has been used in a wide range of studies and has been translated to and validated in Swedish [17, 18]. In short, the instrument consists of 30 items, divided into 15 scales/items: one Global health status/QoL scale (QL2; 2 items); five functional scales: Physical functioning (PF2; 5 items), Role functioning (RF2; 2 items), Emotional functioning (EF; 4 items), Cognitive functioning (CF; 2 items), and Social functioning (SF; 2 items); and nine symptom scales/items: Fatigue (FA; 3 items), Nausea and vomiting (NV; 2 items), Pain (PA; 2 items), Dyspnoea (DY; 1 item), Insomnia (SL; 1 item), Appetite loss (AP; 1 item), Constipation (CO; 1 item), Diarrhoea (DI; 1 item), and Financial difficulties (FI; 1 item). For the functional and symptom items, responses are given on a four-point scale ranging from “Not at all” (1 point) to “Very much” (4 points), while for the Global health status/QoL items a seven-point scale ranging from “Very poor” (1 point) to “Excellent” (7 points) is used. The raw scores for each scale/item are summarized and linearly transformed such that each scale/item ranges from 0 to 100 points. High scores for the Global health status/QoL and functional scales are interpreted as high QoL and high/healthy levels of functioning, respectively, thus corresponding to a high HRQoL, while high scores for symptom scales/items are interpreted as high levels of symptomatology/problems, thus corresponding to a low HRQoL. [22] Data on QLQ-C30 from a random sample of 60–69 years old women (corresponding to the mean age at diagnosis of the women included in the present study) in Sweden (n = 415) given by Derogar et al. [23] were used as normal population reference values for the present study.

### Study variables

Country of birth was self-reported and collected using the study-specific questionnaire, with the following four pre-specified categories available for the respondents to choose from: Sweden, other Nordic country (ONC), other European country (OEC), or non-European country (NEC). As confounding variables, data on education level, cohabiting status, having children living at home, occupational status (employed, studying, on sick leave, retired, or on paternal leave; multiple choices possible), and number of cancer treatment modalities received were collected using the study-specific questionnaire. The latter variable included surgery, radiation therapy, chemotherapy, and other treatment, with multiple choices possible and one point given for each treatment modality, resulting in a total of 0–4 points. Date of birth was obtained from each patient’s PIN, while type of cancer (ICD-10 code), date of diagnosis, and FIGO stage were obtained from the SCR. Age at diagnosis was calculated as the difference between date of birth and date of diagnosis, while time from diagnosis to eligibility was calculated as the difference between the date of diagnosis and the date when the individual was deemed eligible to participate in the study due to still being alive and the research team obtaining a valid address to the woman from the Swedish Population Register.

To take comorbidities into account, data on medical diagnoses from all health care providers in Region Stockholm (including primary as well as inpatient and outpatient care) reported during the 5 years before the date of cancer diagnosis were obtained from the VAL data base. Medical diagnoses were reported as ICD-10 or KSH97-P (*Klassifikation av sjukdomar och hälsoproblem 1997—Primärvård*, Classification of Diseases and Health Problems 1997—Primary Care) codes, the latter being an abbreviated version of ICD-10 adapted to Swedish primary health care [24]. These codes were then used to calculate the Charlson Comorbidity Index (CCI) [25] using the coding algorithm of Quan et al. [26] and the weights given by Charlson et al. [25]. One woman with missing value on CCI was given a value of 0 for this variable.

Finally, data on geodemographic segmentation were obtained from the VAL data base for the years of eligibility and cancer diagnosis. The geodemographic segmentation data provided by VAL were derived using the Swedish version of the Mosaic classification system (InsightOne Nordic AB, Stockholm, Sweden) [27]. This system classified each residential area in Sweden into one of three groups based on demographic, socio-economic, and other important variables: Well-off areas, moderately well-off areas, or less well-off areas. All women participating in the present study were classified into the group corresponding to the area they lived in prior to the date when they were deemed eligible to participate in the study. In the case that the woman had moved from one area to another, the area she lived in closest in time to the date of eligibility was used.

### Statistical analyses

Categorical data are presented as frequencies and percentages, n (%), while ordinal and continuous data are given as means with accompanying standard deviations (SDs). Tests of differences between groups were performed using Pearson’s χ^2^-test for categorical data and the Kruskal–Wallis rank sum test for ordinal, discrete, and continuous data. When the χ^2^-approximation for Pearson’s χ^2^-test was considered to possibly be incorrect, P-values were calculated using the Monte Carlo simulation method of Hope [28] with 1,000,000 replications.

The magnitude of the association between country of birth and HRQoL was estimated using adjusted and unadjusted linear regression models, separately for each of the 15 QLQ-C30 scales/items, with the four groups of country of birth (reference category: Sweden) as main risk factor and age at diagnosis (years), time from diagnosis to eligibility (years), education level (primary level or lower/secondary level/college or university), year of diagnosis, cohabiting (yes/no), having children living at home (yes/no), employed (yes/no), on sick leave (yes/no), retired (yes/no), geodemographic group (well-off area/moderately well-off area/less well-off area), CCI, number of different treatments, type of cancer (cervical/uterine/ovarian/other [vulvar, vaginal, or fallopian tube]), and FIGO stage (I/II/III/IV) as confounding variables in the adjusted analyses. Results are reported as slope coefficient β with 95% confidence intervals (CIs). All statistical analyses were performed in R 4.2.0 (R Foundation for Statistical Computing, Vienna, Austria), with two-sided P-values < 0.05 considered statistically significant.

## Results

Demographic and clinical characteristics for the 684 participating women, according to country of birth, are given in Table 1. In total, the study sample included 555 (81.1%) Swedish-born, 54 (7.9%) ONC-born, 43 (6.3%) OEC-born, and 32 (4.7%) NEC-born women. The participants’ mean (SD) age was 65.4 (14.0) years at the time of diagnosis, with a large majority (n = 425; 62.1%) being ≥ 65 years old. Overall, 359 (52.7%) of the participants completed the study questionnaire online. The most common (n = 256; 37.4%) year of diagnosis was 2016, with a mean (SD) time of 0.96 (0.40) years from diagnosis to being deemed eligible to participate in the study. College/university was the most common education level, attained by 283 (42.1%) of the women. Three out of five (n = 400; 59.2%) women were cohabiting and 15.4% (n = 104) had children living at home. In total, 202 (29.5%) of the participating women were (partly or full-time) employed, while 70 (7.3%) were on sick-leave and 451 (65.9%) were retired. Living in a moderately well-off area was most common (n = 289; 42.3%). Overall, the participating women had a mean (SD) CCI of 1.6 (0.7) points and had received 1.6 (0.7) different treatments. Most women (n = 364; 53.2%) were diagnosed with uterine cancer, and a large majority had FIGO stage I (n = 428; 62.6%).

**Table 1:** Demographic and clinical characteristics for the 684 women in the study population according to country of birth

	Country of birth					
	Total	Sweden	Other Nordic	Other European	Non-European	
	n = 684	n = 555	n = 54	n = 43	n = 32	
Variable		(81.1%)	(7.9%)	(6.3%)	(4.7%)	P-value
Age at diagnosis (years), mean (SD)^a^	65.4 (14.0)	65.8 (14.1)	68.2 (10.0)	61.5 (14.2)	57.9 (15.1)	**0.001**^e^
Age at diagnosis grouped, n (%)						**0.009**^ g^
< 50 years	101 (14.8)	81 (14.6)	3 (5.6)	10 (23.3)	7 (21.9)	
50 to < 65 years	158 (23.1)	120 (21.6)	12 (22.2)	14 (32.6)	12 (37.5)	
≥ 65 years	425 (62.1)	354 (63.8)	39 (72.2)	19 (44.2)	13 (40.6)	
Completing the questionnaire online, n (%)	359 (52.7)	301 (54.5)	29 (53.7)	13 (30.2)	16 (50.0)	**0.023**^ g^
Year of diagnosis, n (%)						**0.029**^f^
2014	185 (27.0)	151 (27.2)	21 (38.9)	7 (16.3)	6 (18.8)	
2016	256 (37.4)	199 (35.9)	21 (38.9)	24 (55.8)	12 (37.5)	
2018	243 (35.5)	205 (36.9)	12 (22.2)	12 (27.9)	14 (43.8)	
Time from diagnosis to eligibility (years), mean (SD)	0.96 (0.40)	0.98 (0.40)	0.88 (0.37)	0.86 (0.41)	0.99 (0.45)	0.120^e^
Education level, n (%)						0.372^f^
Primary or lower	117 (17.4)	86 (15.7)	13 (24.5)	11 (26.2)	7 (22.6)	
Secondary	273 (40.6)	223 (40.8)	22 (41.5)	16 (38.1)	12 (38.7)	
College/university	283 (42.1)	238 (43.5)	18 (34.0)	15 (35.7)	12 (38.7)	
Cohabiting, n (%)	400 (59.2)	328 (59.5)	26 (49.1)	26 (61.9)	20 (66.7)	0.376^f^
Having children living at home, n (%)	104 (15.4)	86 (15.5)	3 (5.6)	6 (14.0)	9 (28.1)	**0.043**^ g^
Employed, n (%)^b^	202 (29.5)	167 (30.1)	10 (18.5)	14 (32.6)	11 (34.4)	0.286^f^
On sick leave, n (%)^b^	50 (7.3)	39 (7.0)	3 (5.6)	6 (14.0)	2 (6.2)	0.367^ g^
Retired, n (%)^b^	451 (65.9)	370 (66.7)	42 (77.8)	22 (51.2)	17 (53.1)	**0.018**^f^
Geodemographic group, n (%)						**0.005**^f^
Well-off area	206 (30.1)	179 (32.3)	12 (22.2)	8 (18.6)	7 (21.9)	
Moderately well-off area	289 (42.3)	240 (43.2)	23 (42.6)	17 (39.5)	9 (28.1)	
Less well-off area	189 (27.6)	136 (24.5)	19 (35.2)	18 (41.9)	16 (50.0)	
Charlson comorbidity index (CCI), mean (SD)	1.6 (2.3)	1.6 (2.3)	1.5 (2.5)	1.7 (2.6)	1.0 (1.8)	0.354^e^
Number of different treatments received, mean (SD)^c^	1.6 (0.7)	1.6 (0.7)	1.5 (0.7)	1.5 (0.6)	1.5 (0.7)	0.920^e^
Type of cancer, n (%)						0.259^ g^
Cervical	114 (16.7)	95 (17.1)	3 (5.6)	9 (20.9)	7 (21.9)	
Ovarian	133 (19.4)	108 (19.5)	12 (22.2)	7 (16.3)	6 (18.8)	
Uterine	364 (53.2)	287 (51.7)	36 (66.7)	25 (58.1)	16 (50.0)	
Other^d^	73 (10.7)	65 (11.7)	3 (5.6)	2 (4.7)	3 (9.4)	
FIGO level, n (%)						0.299^ g^
I	428 (62.6)	348 (62.7)	35 (64.8)	25 (58.1)	20 (62.5)	
II	70 (10.2)	52 (9.4)	6 (11.1)	6 (14.0)	6 (18.8)	
III	130 (19.0)	112 (20.2)	5 (9.3)	9 (20.9)	4 (12.5)	
IV	56 (8.2)	43 (7.7)	8 (14.8)	3 (7.0)	2 (6.2)	

*SD* standard deviation. Out of all women living in Region Stockholm during the years 2014, 2016, and 2018, there were on average 75.6% born in Sweden, 3.5% in other Nordic countries, 7.0% in other European countries, and 13.9% in non-European countries [11]. There were 3 (0.4%) missing values for completing the questionnaire online, 11 (1.6%) missing values for education level, 8 (1.2%) for cohabiting, and 7 (1.0%) for having children living at home, which were excluded from the analyses

Significant P-values are given in bold

^a^Total mean (SD) 65.4 (14.0) years, minimum 21.4 years, median 68.4 years, maximum 93.7 years

^b^Multiple choices possible

^c^Possible range 0–4 points

^d^Includes vulvar, vaginal, and fallopian tube cancer. P-value for comparison between countries of birth calculated using

^e^Kruskal-Wallis rank sum test, ^f^Pearson’s χ^2^-test

^g^Pearson’s χ^2^-test with Monte Carlo simulation

Age at diagnosis differed significantly between the four country groups (P = 0.001), with NEC-born women being the youngest at a mean (SD) age of 57.9 (15.1) years and 40.6% of the individuals in the group being ≥ 65 years old, while ONC-born women were the oldest at a mean (SD) age of 68.2 (10.0) years with 72.2% being ≥ 65 years old (P = 0.009 for age groups). Completing the study questionnaire online was most common among Swedish-born women (n = 301; 54.5%) and least common among OEC-born women (n = 13; 30.2%), with a statistically significant difference between groups (P = 0.023). Likewise, year of diagnosis differed significantly between the four groups (P = 0.029), with 2018 being the most common year for Swedish-born (n = 205; 36.9%) and NEC-born (n = 14; 43.8%) women, while 2016 was the most common year for OEC-born (n = 24; 55.8%) women, and for ONC-born it was tied between 2014 and 2016 (n = 21; 38.9% for both years). Having children living at home was most common among NEC-born (n = 9; 28.1%) and least common among ONC-born (n = 3; 5.6%), with a significant difference between groups (P = 0.043). For occupational status, only retirement status differed significantly between countries of birth (P = 0.018), with the lowest proportion (n = 22; 51.2%) observed for OEC-born and the highest proportion (n = 42; 77.8%) for ONC-born. Finally, geodemographic group also differed significantly between the four country groups (P = 0.005), with living in a moderately well-off area being most common among Swedish-born (n = 240; 43.2%) and ONC-born (n = 23; 42.6%) women, while living in less well-off areas was most common for OEC-born (n = 18; 41.9%) and NEC-born (n = 16; 50.0%) women.

### Differences in health-related quality of life

**Fig. 2:**
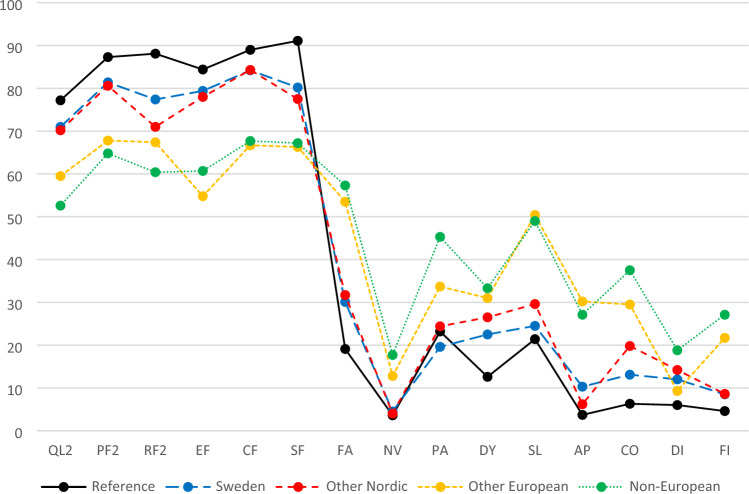
Values for the 15 QLQ-C30 scales/items according to country of birth, as well as for the random sample of 60–69 years old women in Sweden used as normal population reference group. *QL2* Global health status/QoL; *PF2* Physical functioning; *RF2* Role functioning; *EF* Emotional functioning; *CF* Cognitive functioning; *SF* Social functioning; *FA* Fatigue; *NV* Nausea and vomiting; *PA* Pain; *DY* Dyspnoea; *SL* Insomnia; *AP* Appetite loss; *CO* Constipation; *DI* Diarrhoea; *FI* Financial difficulties

Results for HRQoL measured by the 15 QLQ-C30 scales/items according to country of birth, together with reference values for the normal population of 60–69 years old women in Sweden, are given in Table 2 and Fig. 2. Notably, HRQoL differed significantly between the four country groups for 14 of the 15 scales/items, the only exception being diarrhoea. Mostly, Swedish-born women reported higher HRQoL (i.e., higher level of QoL/functioning and lower level of symptomatology/problems), the only exceptions being cognitive functioning, nausea and vomiting, and appetite loss, for which the ONC group reported higher HRQoL. Likewise, NEC-born women reported the lowest HRQoL for all QLQ-C30 scales/items except emotional, cognitive, and social functioning, insomnia, and appetite loss, for which OEC-born women had the lowest HRQoL.

**Table 2:** QLQ-C30 scales/items according to country of birth, together with reference values for 60–69 years old women in Sweden

	Reference values^a^	Country of birth				
		Sweden	Other Nordic	Other European	Non-European	
Scale/item	Mean (SD)	Mean (SD)	Mean (SD)	Mean (SD)	Mean (SD)	P-value^b^
Global health status/QoL (QL2)	77.2 (21.8)	71.0 (21.3)	70.2 (21.7)	59.5 (21.3)	52.6 (25.3)	** < 0.001**
Physical functioning (PF2)	87.3 (16.8)	81.4 (21.5)	80.6 (20.2)	67.8 (23.9)	64.8 (26.5)	** < 0.001**
Role functioning (RF2)	88.1 (22.5)	77.4 (29.4)	71.0 (31.8)	67.4 (30.9)	60.4 (33.5)	**0.001**
Emotional functioning (EF)	84.4 (19.4)	79.4 (21.4)	78.0 (22.9)	54.8 (27.6)	60.7 (27.4)	** < 0.001**
Cognitive functioning (CF)	89.0 (16.0)	84.2 (20.9)	84.3 (18.4)	66.7 (30.4)	67.7 (25.7)	** < 0.001**
Social functioning (SF)	91.1 (18.9)	80.2 (25.7)	77.5 (29.5)	66.3 (34.4)	67.2 (33.5)	**0.014**
Fatigue (FA)	19.1 (22.3)	30.1 (25.7)	31.7 (24.5)	53.5 (29.4)	57.3 (27.2)	** < 0.001**
Nausea and vomiting (NV)	3.6 (9.6)	4.5 (11.5)	4.0 (11.2)	12.8 (18.5)	17.7 (18.4)	** < 0.001**
Pain (PA)	23.2 (27.8)	19.6 (26.5)	24.4 (28.0)	33.7 (31.0)	45.3 (30.3)	** < 0.001**
Dyspnoea (DY)	12.6 (21.8)	22.5 (28.6)	26.5 (26.2)	31.0 (28.5)	33.3 (29.3)	**0.010**
Insomnia (SL)	21.4 (28.1)	24.5 (28.4)	29.6 (27.2)	50.4 (34.4)	49.0 (32.8)	** < 0.001**
Appetite loss (AP)	3.7 (13.8)	10.3 (22.2)	6.2 (16.0)	30.2 (32.4)	27.1 (31.0)	** < 0.001**
Constipation (CO)	6.3 (17.5)	13.1 (23.5)	19.8 (30.0)	29.5 (30.2)	37.5 (36.7)	** < 0.001**
Diarrhoea (DI)	6.0 (17.0)	12.0 (23.0)	14.2 (27.2)	9.3 (22.2)	18.8 (29.3)	0.332
Financial difficulties (FI)	4.6 (15.4)	8.5 (21.4)	8.6 (23.5)	21.7 (28.1)	27.1 (34.3)	** < 0.001**

*QoL* quality of life; *SD* standard deviation. All scales/items have a possible range of 0–100 points

Significant P-values are given in bold

^a^Reference values for 60–69 years old women (n = 415), given by Derogar et al. [23]

^b^P-value for comparison between different countries of birth using the Kruskal–Wallis rank sum test

For the QoL/functioning scales/items, Swedish-born women had on average 2.0, 15.2, and 16.7 points higher values than women in the ONC, OEC, and NEC groups, respectively, while they had 2.2, 14.1, and 18.7 points lower values than those in the ONC, OEC, and NEC groups, respectively, for the symptom scales/items (not in table). Except for pain, all groups of women had lower HRQoL than the reference group of 60–69 years old women in Sweden (Fig. 2).

Results from adjusted and unadjusted linear regression analyses of the association between country of birth and HRQoL, separately for each QLQ-C30 scale/item, are presented in Table 3. Notably, for both adjusted and unadjusted analyses, the ONC-born women did not differ significantly from Swedish-born women for any of the QLQ-C30 scales/items, while NEC-born women differed significantly from Swedish-born women for all QLQ-C30 scales/items except diarrhoea. Likewise, OEC-born women differed significantly from Swedish-born women for all QLQ-C30 scales/items except dyspnoea and diarrhoea, and in the adjusted models also role functioning. In all these cases, both OEC-born and NEC-born women had lower levels of QoL/functioning and higher levels of symptomatology/problems than Swedish-born women. For NEC-born women, the largest difference (95% CI) compared with Swedish-born women in the adjusted analyses was 27.4 (17.9–37.0) points for fatigue, followed by 25.6 (15.3–35.9) points for pain, and 25.4 (14.2–36.6) points for insomnia, with the corresponding values for OEC-born women being 23.9 (14.5–33.2) points for insomnia, −22.6 (−29.6 to −15.6) points for emotional functioning, and 21.7 (13.7 to 29.6) points for fatigue (P < 0.001 for all).

**Table 3:** Results from adjusted and unadjusted linear regression analyses of the association between country of birth and HRQoL, separately for each QLQ-C30 scale/item

		Unadjusted			Adjusted^b^		
Outcome	Country of birth^a^	β	95% CI	P-value	β	95% CI	P-value
Global health status / QoL (QL2)	Other Nordic	−0.8	−6.9 to 5.3	0.797	−2.0	−8.1 to 4.0	0.505
	Other European	−11.5	−.3 to −4.8	** < 0.001**	−10.3	−17.0 to −3.6	**0.002**
	Non-European	−18.4	−26.1 to −10.7	** < 0.001**	−18.6	−26.5 to −10.7	** < 0.001**
Physical functioning (PF2)	Other Nordic	−0.8	−7.0 to 5.3	0.792	0.0	−5.9 to 5.9	1.000
	Other European	−13.7	−20.5 to −6.9	** < 0.001**	−12.5	−19.0 to −6.0	** < 0.001**
	Non-European	−16.6	−24.5 to −8.8	** < 0.001**	−19.5	−27.2 to −11.8	** < 0.001**
Role functioning (RF2)	Other Nordic	−6.4	−14.8 to 2.0	0.133	−6.2	−14.2 to 1.9	0.131
	Other European	−9.9	−19.3 to −0.6	**0.036**	−6.8	−15.7 to 2.2	0.136
	Non-European	−17.0	−27.7 to −6.3	**0.002**	−18.4	−29.0 to −7.8	** < 0.001**
Emotional functioning (EF)	Other Nordic	−1.4	−7.7 to 4.9	0.656	−1.8	−8.1 to 4.5	0.570
	Other European	−24.5	−31.5 to −17.6	** < 0.001**	−22.6	−29.6 to −15.6	** < 0.001**
	Non-European	−18.7	−26.7 to −10.7	** < 0.001**	−15.7	−24.0 to −7.5	** < 0.001**
Cognitive functioning (CF)	Other Nordic	0.0	−6.1 to 6.1	0.994	−0.6	−6.6 to 5.5	0.848
	Other European	−17.6	−24.4 to −10.8	** < 0.001**	−15.8	−22.5 to −9.1	** < 0.001**
	Non-European	−16.5	−24.3 to −8.7	** < 0.001**	−15.8	−23.7 to −7.9	** < 0.001**
Social functioning (SF)	Other Nordic	−2.8	−10.4 to 4.8	0.472	−3.9	−11.1 to 3.4	0.294
	Other European	−14.0	−22.4 to −5.5	**0.001**	−11.3	−19.3 to −3.2	**0.006**
	Non-European	−13.1	−22.7 to −3.4	**0.008**	−15.3	−25.0 to −5.6	**0.002**
Fatigue (FA)	Other Nordic	1.6	−5.7 to 8.9	0.666	1.1	−6.1 to 8.2	0.767
	Other European	23.4	15.3 to 31.5	** < 0.001**	21.7	13.7 to 29.6	** < 0.001**
	Non-European	27.2	17.9 to 36.5	** < 0.001**	27.4	17.9 to 37.0	** < 0.001**
Nausea and vomiting (NV)	Other Nordic	−0.5	−4.0 to 3.0	0.767	−0.6	−4.2 to 3.0	0.742
	Other European	8.3	4.4 to 12.2	** < 0.001**	7.2	3.2 to 11.2	** < 0.001**
	Non-European	13.2	8.7 to 17.6	** < 0.001**	10.8	6.0 to 15.6	** < 0.001**
Pain (PA)	Other Nordic	4.7	−2.9 to 12.4	0.220	5.5	−2.2 to 13.3	0.158
	Other European	14.1	5.6 to 22.6	**0.001**	11.5	2.9 to 20.1	**0.008**
	Non-European	25.7	16.0 to 35.4	** < 0.001**	25.6	15.3 to 35.9	** < 0.001**
Dyspnoea (DY)	Other Nordic	4.1	−3.9 to 12.1	0.315	4.0	−3.9 to 12.0	0.314
	Other European	8.5	−0.3 to 17.4	0.058	7.5	−1.4 to 16.3	0.095
	Non-European	10.9	0.7 to 21.1	**0.036**	16.7	6.1 to 27.3	**0.002**
Insomnia (SL)	Other Nordic	5.1	−3.0 to 13.3	0.214	5.9	−2.5 to 14.4	0.166
	Other European	25.9	16.8 to 34.9	** < 0.001**	23.9	14.5 to 33.2	** < 0.001**
	Non-European	24.5	14.1 to 34.8	** < 0.001**	25.4	14.2 to 36.6	** < 0.001**
Appetite loss (AP)	Other Nordic	−4.2	−10.6 to 2.3	0.205	−4.3	−10.8 to 2.2	0.189
	Other European	19.9	12.7 to 27.1	** < 0.001**	18.3	11.1 to 25.6	** < 0.001**
	Non-European	16.8	8.5 to 25.0	** < 0.001**	19.1	10.5 to 27.8	** < 0.001**
Constipation (CO)	Other Nordic	6.7	−0.4 to 13.8	0.064	4.5	−2.7 to 11.7	0.220
	Other European	16.4	8.5 to 24.3	** < 0.001**	14.7	6.7 to 22.6	** < 0.001**
	Non-European	24.4	15.4 to 33.5	** < 0.001**	24.5	14.9 to 34.0	** < 0.001**
Diarrhoea (DI)	Other Nordic	2.2	−4.5 to 8.8	0.517	1.5	−5.2 to 8.2	0.648
	Other European	−2.7	−10.1 to 4.7	0.469	−3.9	−11.4 to 3.5	0.297
	Non-European	6.7	−1.7 to 15.2	0.117	8.6	−0.3 to 17.5	0.057
Financial difficulties (FI)	Other Nordic	0.1	−6.3 to 6.5	0.972	0.3	−5.6 to 6.2	0.921
	Other European	13.2	6.1 to 20.3	** < 0.001**	9.7	3.1 to 16.3	**0.004**
	Non-European	18.6	10.4 to 26.7	** < 0.001**	11.9	4.0 to 19.8	**0.003**

*CI* confidence interval; *QoL* quality of life. All outcome scales/items have a possible range of 0–100 points

Significant P-values are given in bold

^a^Reference category: Sweden

^b^Adjusted for age at diagnosis (years), time from diagnosis to eligibility (years), education level (primary level or lower/secondary level/college or university), year of diagnosis, cohabiting (yes/no), having children living at home (yes/no), employed (yes/no), on sick leave (yes/no), retired (yes/no), geodemographic group (well-off area/moderately well-off area/less well-off area), Charlson comorbidity index, number of different treatments received (0–4 points), type of cancer [cervical/uterine/ovarian/other (vulvar, vaginal, or fallopian tube)], and FIGO stage (I/II/III/IV)

R^2^-values (%): QL2 15.0; PF2 22.7; RF2 19.9; EF 17.4; CF 17.0; SF 20.8; FA 21.2; NV 10.8; PA 11.8; DY 12.3; SL 11.3; AP 16.2; CO 12.2; DI 9.8; FI 24.2

Finally, it should be noted that for the confounding clinical variables CCI and FIGO stage, there were in most cases no significant association with HRQoL in the adjusted analyses. For CCI there was thus a significant association only for physical functioning, role functioning, insomnia, and appetite loss, while for FIGO stage there was a significant association only for social functioning, dyspnoea, appetite loss, and diarrhoea (values not shown).

## Discussion

The present study, utilizing patient-reported and register data, found statistically significant differences in HRQoL between native and foreign-born gynaecological cancer patients in Region Stockholm, Sweden. Our results showed that Swedish-born women in general reported the highest HRQoL, with the highest level of QoL/functioning and lowest level of symptomatology/problems, followed in turn by ONC-, OEC-, and NEC-born women. The differences between Swedish-born and ONC-born women were, however, small and non-significant. For OEC- and NEC-born women were, on the other hand, the differences in HRQoL on average quite large (15.2– 16.7 points for QoL/functioning scales/items and 14.1−18.7 points for symptom scales/items), and in most cases strongly statistically significant. The overall conclusion is that the larger the geographic distance of the woman’s country of birth is to that of the Swedish-born women, the lower is the woman’s HRQoL.

### Results in perspective

The association between geographic origin and HRQoL reported in the present study should be viewed in relation to residential segregation, which has been reported as a salient feature of all larger Swedish cities [29] and often referred to when discussing socio-economic inequalities related to health aspects such as cancer. For example, a recent Swedish report showed that living in deprived areas was associated with a lower cervical cancer screening uptake [30]. These results are in line with previous research from other countries. In a French study using population-based data, Poiseuil et al. showed that socioeconomic factors were associated with net survival among breast and gynaecological cancer patients [31]. Another study from France showed that a number of barriers to cervical screening attendance were associated with low socioeconomic status [32]. Previous research on women’s awareness of symptoms related to gynaecological cancer has also shown that lower health literacy, which in turn is more common among women with lower socioeconomic status, is associated with a lower awareness of gynaecological cancer symptoms, [33] adding yet another explanation to why these women tend to seek medical care at a later stage.

Most studies have, however, focused on differences in mortality rates [31, 34]. Sassenou et al. [35] concluded that obese women were less likely to undergo cervical cancer screening and at the same time more often lived in areas with low socioeconomic status, thus being particularly vulnerable. Rauh-Hain et al. [36] pointed to disparities in the treatment of gynaecological cancer related to ethnicity, together with higher cancer-specific mortality.

Disparities in HRQoL level between native and foreign-born individuals have been found in several population-based studies. Earlier studies in Sweden have reported that foreign-born were more likely to report lower HRQoL levels than native-born Swedes [37–39]. Similar differences have been found in studies from other European countries [40–43], where immigration-related factors such as low levels of integration, low education level, and older age at immigration were found to be associated with lower HRQoL levels. A recent study using data from the Swedish Pregnancy Register reported that women born in Latin America, South Asia, North Africa, and the Middle East reported poorer health compared to women born in Sweden [44]. Poor self-reported health has also been reported as a strong risk factor for later mortality and morbidity [45, 46].

### Clinical implications

The risk for low HRQoL among foreign-born women should lead to a high awareness among healthcare personnel when planning support and symptom control during and after cancer treatment. Interestingly, results from a previous study from the Stockholm region, including partly the same data set as used in the present study, showed that some foreign-born cancer (including gynaecological cancer) patients were more likely to gain access to supportive care (e.g., individual care plans and support from a contact nurse) compared with Swedish-born patients [15]. The authors suggested that this might reflect strategies from the healthcare personnel of providing extra support to patients they deemed might need it the most due to socioeconomic vulnerabilities that might influence their HRQoL. Therefore, it is important that patients with cancer are identified in an early stage, thus enabling healthcare personnel to prepare providing appropriate support early on. For patients with gynaecological cancer, this support could for instance include individual rehabilitation strategies, extra support in self-management, and psychosocial support.

### Strengths and limitations

A major strength of the present study was the population-based approach, aiming to include all eligible women in the HCR diagnosed with gynaecological cancer during the study period, thus increasing the generalizability of the study. Another strength was the division of the foreign-born group into three distinct categories, providing a crude measure of cultural, societal, and linguistic distance to the Swedish-born participants and thus making it possible to distinguish different levels of HRQoL in the heterogeneous group of foreign-born gynaecological cancer patients living in Sweden. Moreover, the inclusion of data on the severity of the disease as well as comorbidities made it possible to adjust for factors that may have an important influence on patients’ HRQoL.

A limitation was, however, that no information was available on how long the participating women had lived in Sweden or how fluent they were in Swedish. Moreover, despite the proportion of foreign-born in this study being 18.9%, it must be assumed that those with a limited understanding of Swedish participated in the study to a lower degree. The latter group will to a larger degree consist of individuals with more recent permits to stay in Sweden, and the generalizability of the results of the present study to this group is thus limited. Previous research has even found that this group in some cases may have a higher QoL than sex- and age-matched Swedish-born individuals [47]. Another limitation was the use of self-reported country of birth collected using the study-specific questionnaire, with information about country of birth thus not being available for all potential participants, making it impossible to calculate the response rate according to country of birth. A limitation of using the geodemographic segmentation variable was that the exact methods and data used to construct this variable was not available, thus in some ways making it work as a *black box*. Finally, it should be noted that the design of the study, with HRQoL measured the year after the diagnosis, limits the generalizability of the results since women with more aggressive forms of cancer, with short survival time, could be expected to have participated in the study to a lesser degree. These women could also be expected to, overall, have a lower HRQoL.

## Conclusions

HRQoL differs between native and foreign-born gynaecological cancer patients in Sweden, with Swedish-born women in general having the highest HRQoL. The larger the geographic distance of the woman’s country of birth is to that of the Swedish-born women, the lower is the woman’s HRQoL. These findings could contribute to more individualised cancer care planning, with tailored support to optimize HRQoL for this vulnerable group of patients, diagnosed and treated for gynaecological cancer. A key contribution of the present study is that, to be able to reduce unmotivated differences in HRQoL among women with gynecological cancer, health care professionals need to have specific knowledge about HRQOL and which factors affect it, to be able to systematically identify patients particularly at risk. We suggest person-centered support to address these issues.

## Abbreviations

CI: Confidence interval
EORTC: European Organisation for Research and Treatment of Cancer
HCR: Health care region
HRQoL: Health-related quality of life
ICD-10: International Statistical Classification of Diseases and Related Health Problems, 10th revision
NEC: Non-European country
OEC: Other European country
ONC: Other Nordic country
PIN: Personal Identification Number
QLQ: Quality of life questionnaire
QoL: Quality of life
SCR: Swedish Cancer Register
SD: Standard deviation
